# Preserve and strengthen family to promote mental health

**DOI:** 10.4103/0019-5545.64582

**Published:** 2010

**Authors:** Ajit Avasthi

**Affiliations:** Department of Psychiatry, Postgraduate Institute of Medical Education and Research, Chandigarh, India

## INTRODUCTION

Unlike the West, in India, family is the key resource in the care of patients with mental illness. Families assume the role of primary caregivers for two reasons. First, it is because of the Indian tradition of interdependence and concern for near and dear ones in adversities. Due to this most Indian families prefer to be meaningfully involved in all aspects of care of their relatives despite it being time-consuming. Second, there is a paucity of trained mental health professionals required to cater to the vast majority of the population; hence, the clinicians depend on the family. Thus, having an adequate family support is the need of the patient, clinician and the healthcare administrators.

The term family has its root in the Latin word ‘familia’ that denotes a household establishment, akin to ‘famulus’, which denoted a servant who came from that household establishment. In the ancient Roman law, the word denoted the group of producers, slaves and other servants as well as members connected by common descent or marriage. Family as we understand today has been defined in the Oxford dictionary as (i) The body of persons who live in one house or under one head, including parents, children, servants, (ii) The group consisting of parents and their children, whether living together or not; in wider sense, all those who are nearly connected by blood or affinity. (iii) A person’s children reared collectively. (iv) Those descended, or claiming descent from a common ancestor. From the point of view of psychiatry, family denotes a group of individuals who live together during important phases of their life time and are bound to each other by biological and /or social and psychological relationship. It is a group defined by a sexual relationship sufficiently precise and enduring to provide for the procreation and upbringing of children.[1]

When we look at the family as a unit, the following features are common across the globe: it is universal, permanent, nucleus of all social relationships, has an emotional basis, has a formative influence over its members, teaches its members as to what is their social responsibility and the necessity for co-operation and follows a social regulation.[2]

## FEATURES OF TRADITIONAL INDIAN FAMILIES

India is a secular and pluralistic society characterized by tremendous cultural and ethnic diversity. In India the family is the most important institution that has survived through the ages. India, like most other less industrialized, traditional, eastern societies is a collectivist (a sense of harmony, interdependence and concern for others) society that emphasizes family integrity, family loyalty, and family unity. More specifically, collectivism is reflected in greater readiness to cooperate with family members and extended kin on decisions affecting most aspects of life, including career choice, mate selection, and marriage.[3]

Since ages, the Indian family has been a dominant institution in the life of individuals. It is considered strong, stable, close, resilient and enduring. In India, overwhelmingly, families adhere to a patriarchal ideology, follow the patrilineal rule of descent, are patrilocal, have familialistic value orientations, and endorse traditional gender role preferences. Historically, the traditional, ideal and desired family in India is the joint family. A joint family includes kinsmen, and generally includes three to four living generations, including uncles, aunts, nieces, nephews, and grandparents living together in the same household. Frequently, a large joint family divides after the demise of elderly parents, when there is no longer a single authority figure to hold the family together. After division, each new residential unit, in its turn, usually becomes a joint family when sons of the family marry and bring their wives to live in the family home. The lines of hierarchy and authority are clearly drawn, shaping structurally and psychologically complex family relationships. Ideals of conduct are aimed at creating and maintaining family harmony. Women are especially strongly socialized to accept a position subservient to males, to control their sexual impulses, and to subordinate their personal preferences to the needs of the family and kin group. Reciprocally, those in authority accept responsibility for meeting the needs of others in the family group. Psychologically, family members feel an intense emotional interdependence with each other and there is strong interpersonal empathy, closeness, loyalty, and interdependency.[3]

## INTEGRATION OF FAMILY IN MENTAL HEALTH DELIVERY :INDIAN SCENARIO

Until the arrival of the Britishers, there were no organized modern mental healthcare services in India and the mentally ill were looked after by their families or in religious institutions or simply roamed free. The Britishers established ‘mental asylums’ – institutions which were popular in the European countries, where the community felt safe to keep the unwanted, dangerous mentally ill in closed institutions away from family and society. This was initially for their soldiers but the benefits were gradually extended to the Indian population as well. The first mental asylum was established in Bombay in 1745, the second in Calcutta (1781), the third in Madras (1794) and the fourth in Monghyr, Bihar (1795). Around the same time, Philippe Pinel (1745-1826) in France, William Tuke (1732-1822) in England and Benjamin Rush (1745-1813) in United States ushered in the era of ‘moral treatment’ in psychiatry, which included humane care, avoiding physical restraints, better staff patient interaction and an open door system. Adolf Meyer, in 1909, advocated management of mentally ill patients outside the institutions and proposed a comprehensive ‘community mental health approach’ in which psychiatrists, family physicians, police, teachers and social workers would work together to organize primary, secondary, and tertiary preventive measures in the community. All these changes, taking place in Europe and America did not make any impact on the Indian scene. Till 1946, the approach of the Government was to establish custodial and no therapeutic centers, for a small percentage of severely mentally ill and handicapped individuals.[4]

The community mental health movement in USA had its rise and fall between 1950s and 1980s. President John F Kennedy passed a resolution in 1963 to establish community mental health centers and offer care for mentally ill, who would get released from mental hospitals. A large number of community mental health centers were established. But over a period of time, the community psychiatric approach fell into disrepute because severely ill patients who were released did not go to the community mental health centers. They were not accepted by their families, which resulted in ‘trans-institutionalization’. They were housed in private nursing homes, and board-and-care institutions.

In India there has been a long tradition of involving families in the treatment of mentally ill relatives. In 1957, Dr. Vidya Sagar, the then superintendent of Amritsar Mental Hospital, involved the family members of the mentally ill in the management, by allowing them to stay with their patients in open tents pitched in the hospital campus. He showed that the patients recovered fast and were taken back home. Based on this principle, family wards were established in Christian Medical College, Vellore. The benefits were fast recovery, low relapse rates, and family members served as change agents in their community as they identified other patients and guided their family members to approach psychiatric centers for help.[5] This system of utilizing the family in the care of the patient had the additional advantage of relieving the psychiatrically trained staff, particularly nurses and attendants, from routine duties.[6] However, the hospital-based, resource-intensive and infrastructure dependent nature of such programs meant that they were not the most appropriate models to adopt.[7] It was suggested that some culture specific characteristics should be included in the rehabilitation programs to make them more successful in the Indian context e.g. focusing primarily on families of patients, supporting them, helping them cope and easing their burden.[8]

Nearly four decades ago, Bhaskaran observed that more than 75% of the patients living in mental hospitals had no contact with any family member.[9] He reported that the burden of care for a chronic illness, the reduced work output of the patient and the stigma attached to mental illness were the main reasons for the "unwanted patient". Gupta *et al*.[10] report that although 70% of the patients in the Agra Mental Hospital had one or more family members, more than half of them did not have a single visit from a relative in the previous two years. Surveys of the mental hospitals have also shown that large numbers of long-stay patients have practically no contact with the family.[11]

Though the General hospital psychiatric units (GHPUs) were started at Bombay and Calcutta way back in 1933, more and more such units and departments started working in 1960s and 1970s. The GHPUs had a number of advantages over the mental hospitals – they were easily approachable without stigma, they encouraged more outpatient care; they attracted more patients with minor mental health problems and helped in the integration of psychiatry into the general health system. Further, from family point of view, the patient always stayed with the family and the family was intimately involved in the care of mentally ill subjects.[4] It is important to remember that over the years with the increase in GHPUs and private sector psychiatric nursing homes and clinics resulting in availability of better treatment options, more and more subjects are treated in their family setup on both outpatient and in-patient basis. This fact is more amply revealed by huge number of research publications based on the various aspects of care giving in various psychiatric disorders. Further, when one talks to these caregivers, it becomes amply clear that the family considers sending the patient to a mental hospital as a last resort of management.

The decade of 1970s also saw the primary care approach in the area of mental health.[12] The World Health Organization (WHO) brought out a technical report in 1974 and paved the way for community-mental health program.[13] The noted feasibility studies were conducted in and around Sakalwara village, near Bangalore and Raipur Rani block of Ambala district, Haryana.[14,15] Sixty eight experts from the field of mental health, general health and health administration designed the first draft of National Mental Health Program and it was implemented in the country in 1982 with the purpose of promoting community participation in the organization of the services.[16]

## FAMILY: ADVANTAGES FROM MENTAL HEALTH PERSPECTIVE

The traditional joint family that exists in India is seen as a source of social and economic support and is known for its tolerance of deviant behavior and capacity to absorb additional roles in times of crisis.[17,18] Leff *et al*.[19] have suggested that traditional joint families allow for diffusion of burden in families caring for the mentally ill and could be responsible for mediating the good course and outcome of major mental disorders. Reviews of the role of the family in relation to mental health have found that the nuclear family structure is more likely to be associated with psychiatric disorders than the joint family.[17,20] Chandrashekar *et al*.[21] reported that fewer patients from rural families sought hospitalization when compared to urban families because of the existing joint family structure. Gopinath *et al*.[22] reported that patients who hailed from larger families and had a better educational status tended to discontinue attending a day hospital facility within three months. Studies have shown that the larger the family, in terms of it being an extended or joint family, the more it was able to compensate for a dysfunctional member in terms of having fewer expectations.[23] A study by Sharma *et al*.[24] compared schizophrenia patients living in Liverpool, UK and Bangalore, India and found that Bangalore patients were more socially integrated than Liverpool patients, who appeared socially marginalized.

Three large-scale international collaborative studies conducted by the WHO-the International Pilot Study on Schizophrenia (IPSS), the Determinants of Outcome of Severe Mental Disorders (DOSMeD) and the International Study of Schizophrenia (ISoS) – convincingly demonstrated that persons with schizophrenia did better in India and other developing countries, when compared to their Western counterparts and much of this has been attributed to good family support these patients enjoyed in developing countries.[25] Thara *et al*.[26] and Eaton *et al*.[27] while reporting on a 10-year follow-up of first-episode Indian patients, have also highlighted the importance of early therapeutic interventions, including family interventions, by demonstrating that the prevalence of positive and negative symptoms in schizophrenia stabilized in the first two years of the illness.

In India, traditional joint family structures, where family members stay together with their spouses and children, have been significantly replaced in urban areas by “new order" nuclear families. More importantly, the family system has become a highly differentiated and heterogeneous social entity in terms of structure, pattern, role relationships, obligations and values. In the context of the transitional changes in the Chinese society, Pearson and Lam[28] observed that "in countries with low income levels and numerous existential stressors, changes in family structure may make the care giving burden even more onerous".

## ROLE OF FAMILY IN CURRENT MENTAL HEALTH SCENARIO IN INDIA

The shift to community-based psychiatric services has formalized the role of the caregiver. The family and caregiver’s role has been thus integrated in the treatment plans and in policy making. The role of family becomes even greater in a country like ours with more than one billion people where there is a paucity of trained personnel, with the number of mental health professionals not exceeding 5000. For such a huge population, both settings and service providers are grossly inadequate.[29] It is needless to say that a large part of the mental health care takes place in the community making the family as the primary care provider. This scenario is quite different from what happened in the West, where the locus of care shifted to the community only as a result of the deinstitutionalization movement. Figures with respect to how many patients with schizophrenia stay integrated with their families in India vis-à-vis developed countries like the United Kingdom (UK) and United States of America (USA) speak volumes regarding importance of families in the care of such patients. Two cross-cultural studies reported that less than 50% of patients in the Western world lived with their families, while the comparable figure in India was 98.3%.[24,30]

In view of the dismal state of mental health infrastructure in India, expecting community care by a team, as in the developed countries, including an array of trained nurses, rehabilitation specialists, cognitive therapists, social workers, occupational therapists and psychiatrists would be impractical. Therefore, in a country like India, the term "community care" often translates into patients remaining outside hospitals, but with their families. So, it appears that the locus of care will continue to be with the family.

## IMPACT ON FAMILY :THE SILENT SUFFERERS

It is important to recognize that the caregivers/family members also pay a huge price to care for their ill relatives. The impact of mental illness on the caregivers has been evaluated in the form of burden, caregiving experience, psychiatric/ psychological morbidity, coping, financial burden, needs, stigma, etc.

"Burden of care" is defined as "the presence of problems, difficulties or adverse events which affect the life (lives) of the psychiatric patients’ significant others (e.g. members of the household and/or the family)".[31] The concept of ‘burden of care’ has two distinct components - the objective and the subjective as proposed by Hoenig and Hamilton.[32] Objective burden encompasses measurable effects in household disruptions, economic burden, caregivers’ loss of work, social, and leisure roles, and time spent negotiating the mental health, medical, social welfare, and sometimes criminal justice systems. In contrast, subjective burden is the caregiver’s own perception of the impact of caring. It consists of the negative psychological impact on the caregiver and includes feelings of loss, depression, anxiety, anger, sorrow, hatred, uncertainty, guilt, shame or embarrassment, all of which result in much distress and suffering.[33–35] Findings of numerous studies across a range of psychiatric disorders indicate that carers of patients with schizophrenia have the highest negative impact, where a substantial majority (30%- 60%) of caregivers suffer significant distress.[36] These studies have also identified the major areas of (objective) burden, namely adverse effects on the household routine including care of children, disruption of relations within and outside the family, restriction of leisure time activities of caregivers, the strains placed on their finances and employment, the difficulties in dealing with dysfunctional and problem behavior faced by caregivers, and the impact on the mental and physical wellbeing of the carers.[33,36–40] The prevalence of subjective psychological distress has also been found to be very high, with 29 to 60% of the caregivers judged to be suffering from diagnosable psychiatric disorders across different studies which have used the General Health Questionnaire or similar criteria.[36]

Negative caregiving appraisals are associated with higher levels of social impairment and disability, a smaller social network in patients, and with symptoms such as anxiety or depression. However, caregiving not necessarily always has a negative impact. There has been much less research on positive caregiving appraisals. In psychosis, positive caregiving appraisals have been associated with employed patients whose social functioning is better, patients with shorter duration of illness, and caregivers who are more educated and have access to higher levels of social support.[41,42] It has been reported that some of the caregivers may feel a sense of satisfaction or gratification.

Studies from India have shown that the burden of care of schizophrenia is either similar to or even more than chronic physical disorders.[43,44] The amount of burden in schizophrenia has more effect on family than the financial burden.[45,46] Other studies suggest that family burden in other psychiatric disorders e.g. bipolar disorder, obsessive compulsive disorder, substance dependence etc is also comparable to schizophrenia.[47–50] Studies have reported significant burden among caregivers of patients with Alzheimer’s disease where majority of the caregivers experienced significant deterioration in their mental health.[51] Indian studies have reported mixed findings with respect to significant impact of the care giving role on the relatives’ emotional and physical health. Some studies have reported high level of psychiatric morbidity among family carers, while others have observed that, despite high levels of burden, caregivers reported subjective well-being scores in the normal range.[42,52] It has also been found that spouses often take over the breadwinner’s role, and other family members, especially children, take over the index patient’s responsibilities.[53,54]

There are very few studies which have evaluated the cost of mental illnesses in India.[55–60] Studies have shown that 95% of the cost of treatment of schizophrenia is borne by the family, which uses about half of its income in the patient’s treatment.[58] Studies have also shown that cost of treatment of schizophrenia is similar to the cost of treatment of diabetes mellitus but slightly less than the cost of treatment of bipolar disorders.[58,59]

Coping strategies of caregivers have been distinguished into two broad groups: problem-focused and emotion-focused strategies. Problem-focused strategies refer to constructive coping efforts undertaken to modify difficult situations and include measures such as problem-solving, seeking information, or using positive methods of communication. In contrast, the less adaptive emotion-focused strategies are attempts at modulating the caregiver’s stress-related emotional response by measures such as avoiding or resigning themselves to the situation.[36,39,61,62] The most consistent correlates of coping across quite a few studies have been caregiver-burden, patient’s social functioning, expressed emotions (EE) of caregivers and social support available for caregivers. High levels of burden, dysfunction, and EE together with low levels of available support have been associated with a number of maladaptive, principally emotion-focused styles such as avoidance, resignation, coercion, etc.[63–66] Indian studies have shown that use of problem-focused coping, seeking social support as a coping strategy have been found to be related to positive caregiving experience.[67] Another study shows that the coping patterns of caregivers of schizophrenia and bipolar disorders are quite alike, though caregivers of patients with schizophrenia used some of the emotion-focused strategies more often than the caregivers of bipolar subjects. It is also reported that the caregiver’s gender, patient’s level of dysfunction and caregiver-neuroticism have a significant influence on coping patterns.[68]

As the treatment in the West is patient-centric, many studies from there have evaluated the needs of patients with schizophrenia and the mean number of needs reported by patients has varied from 5.3 to 7.9.[69,70] As per the type of needs, most consistently reported area of needs by the patients in various studies include need for company, food, information, house upkeep/looking after home, daytime activities, psychological distress and intimate relations.[69–74] Studies have also consistently shown that mental health services in the West are unable to meet many of these needs of caregivers. This is contributing to burden and dissatisfaction among caregivers, and resultant decreased use of the available services.[75,76]

In a recent study from India, Kulhara *et al*. evaluated the needs of the patients of schizophrenia as perceived by the patients and their family members, and reported that the number of needs of patients as perceived by the caregivers was similar to that perceived by patient’s themselves and western data.[77] However, in contrast to the West, where most of the needs of the patients are met, more than two-third needs of schizophrenia patients in Indian setting were unmet. The most commonly reported needs by both patients and their caregivers were need for welfare benefits. Similarly other studies have also shown that the subjects with severe mental illnesses have unmet treatment and rehabilitation needs, need for meaningful employment, or productive activity, the need for practical and emotional support, the need for information about schizophrenia.[29,78–80] The findings related to employment, income generation and productive activities are not surprising in a low-income country with negligible welfare and social security benefits, since caring for a non-contributory member adds to the already heavy burden of existential stressors. A recent unpublished study found that in the Indian setting most of the needs in schizophrenia and bipolar patients are met by their family members with minimal help from formal sources.[81] In keeping with trends in the Western literature, family carers from India also have concerns about the well-being of their patient after their lifetime, and want these issues to be addressed by professionals.[52,82,83] However, relatives have been less concerned about information regarding the illness, guidance in dealing with patient etc.[80,83,84]

It is well known that stigma affects life of a person with mental illness negatively. It also negatively impacts their family members and relatives. In contrast to the considerable amount of data available on stigmatization of patients, there has been limited exploration of family stigma.[85] Nevertheless, certain themes are evident from research among caregivers of patients with mental illnesses. Family stigma contains the stereotypes of blame, shame, and contamination. Typically, blame is attributed to poor parenting skills, which supposedly lead to mental illness. In turn, family members may experience shame for being blamed for the mental illness. This shame may lead to family members avoiding contact with neighbours and friends. Contamination describes how close association with the stigmatized person might lead to diminished self-worth. Other elements and negative consequences of stigma such as restricted access to all kinds of facilities including healthcare services and discrimination are also shared by patients and caregivers.[86–88] Concerns have been shown about stigma affecting the chances of marriage of the patient, or another member of the family.

## WHY DOES FAMILY CARE FOR THEIR ILL RELATIVES?

A seminal study by Guberman *et al*.[89] interviewed 40 women caregivers of frail elderly or mentally ill relatives and identified 14 factors, which motivated these women to assume the care giving role. The first and most important group of such factors was associated with the caregiver’s material, social, and psychological situation. These included her feelings of closeness and interconnectedness with family, gender-role conditioning, and life situation. These were further elaborated as love, maternal feelings, feelings of family ties, feelings of obligation, resignation and guilt, a profound need to help others, socioeconomic dependence, belief in the healing process, religious or anti-institutional convictions, personal characteristics, and family tradition. An old study which assessed families of 60 patients with chronic schizophrenia undergoing treatment with drug and social therapies found that many of these families maintained an active interest in the patients and expressed their desire to continue visits to the hospital. Their attitude towards the patient’s illness was optimistic and many families favored discharge. Their expectations were realistic and in accord with patient’s capacities.[90] Further, some studies have also reported experiences of gains in caregivers of schizophrenia. In a study, about 70% of the caregivers reported that they had become more sensitive to persons with disabilities. More than 50% reported that caring for their relative helped them greatly to clarify their priorities in life and engendered a greater sense of inner strength.[91]

## ROLE OF FAMILY IN MENTAL DISORDER: CAUSATIVE, PROPAGATIVE, PRECIPITATIVE AND EVOLUTIONARY ROLES

Family has attracted the attention of various researchers in the area of psychiatry to determine the "mysterious" etiopathogenesis of various behavioral disorders. The structure, size, socio-economic status of family, the state of emotional health of family members, education, religion and migratory status of families have been observed to be related to various specific psychiatric illnesses. In this context, researchers have also tried to define dysfunctional families. A dysfunctional family is a family, in which conflict, misbehavior and even abuse on the part of individual members of the family occur continually and regularly, leading other members to accommodate such actions. Dysfunctional families are most often a result of the alcoholism, substance abuse, or other addictions of parents, parents’ untreated mental illness or personality disorders, or the parents emulating their own dysfunctional parents and dysfunctional family experiences. Symptoms and signs of family dysfunction include inconsistency and unpredictability, role reversals ("parentifying" children), "closed family system" (a socially isolated family that discourages relationships with outsiders), "dogmatic or chaotic parenting" (harsh and inflexible discipline), depriving parents (parents who control by withholding love, money, praise, attention, or anything else their child needs or wants), stifled speech (children not allowed to dissent or question authority).

Fromm-Reichmann’s (1948) theory of ‘schizophrenogenic mother’ stated that schizophrenia is due to unconscious rejection of mother for her child, whereas according to the ‘Double Bind Hypothesis’, conflicting communication within the family creates schizophrenia, which is seen as a learned communication pattern. Schizophrenia was believed to be due to living in families in which there is severe emotional conflict (Marital Schism and Skew) or caused by communication problems in family (Transactional Thought Disorder).

A review of studies reveals that psychoneurotic and depressed patients are overrepresented in the unitary and small-sized families, whereas hysteria is observed more commonly in females from joint families. The reason being that in a unitary family there is lesser dilution and fewer opportunities for sharing of emotion, particularly in times of stress which leads to swelling of emotions, in turn leading to formation of a nidus for subsequent precipitation in the form of depression. On the other hand, in the ‘restrictive’ environment of the joint family, women are expected to observe more restraint, all must be subject to command of the ‘elders’, which leads to interpersonal maladjustment. Hysterical manifestations may arise or may get perpetuated because of easy availability of a secondary gain. The upsurge of broken marriages in western culture has been reflected in ever increasing problems of juvenile delinquency, adult aggression, suicide, and drug addiction.[92]

Maladaptive parental behavior is associated with an increased risk for the development of psychiatric disorders among the offspring of parents with and without psychiatric disorders.[93] Psychiatric disorders increased markedly in offspring as the number of maladaptive parental behavior increased. Persistent maladaptive parental behavior was associated with a higher risk of psychiatric disorders in offspring than was episodic maladaptive parental behavior. Maladaptive parental behavior is associated with an increased risk in offspring for anxiety, depression, disruptive personality, and substance use disorders during late adolescence and early adulthood. It has been shown that parental rearing styles exert a very powerful influence on the development of the child. Here it is the perception of the parental behavior by the child that really matters. Rejection of the child and lack of warmth provoke feelings of isolation and a lack of self-esteem and may lead to antisocial behavior.[94,95] In contrast, authoritative-reciprocal parenting as described by Baumrind, contributes to better self-esteem, social responsibility, and academic performance.[96,97]

Childhood exposure to parental verbal aggression is associated, by itself, with moderate to large effects on measures of dissociation, irritability, depression and anger-hostility. This raises the possibility that exposure to verbal aggression may be a stressor that affects the development of certain vulnerable brain regions in susceptible individuals, resulting in psychiatric sequelae. Alternatively, exposure to verbal aggression in childhood may put into force a powerful negative model for interpersonal communication, which is then incorporated as a behavioral response in future relationships.[98]

Children of alcoholics have been found to be at increased risk for various childhood stressors such as abuse, neglect, witnessing domestic violence, or growing up with other forms of household dysfunction. It has been estimated that 30% of child abuse cases involved alcoholic parents, and 60% of domestic violence cases have occurred when the perpetrator was under the influence of alcohol.[99] Family dysfunction is associated with thinking and planning suicide, deliberate self harm, suicide attempts, as well as severity of depression in depressed adolescents.[100]

Another important construct defined in relation to family interactions is called "expressed emotion", which describes the level of criticism, hostility, and emotional over involvement in a family. Although initially developed for patients with schizophrenia and their families, expressed emotion is now studied extensively across the health care spectrum and in many different cultures. High expressed emotion is a "significant and robust" predictor of relapse in many illnesses, such as schizophrenia, depressive disorders, acute mania, and alcoholism. Criticalness is one of the essential components of expressed emotion and is associated with the poorest patient outcomes.[101,102] In a follow-up study in bipolar patients, low level of social support and poor quality of relationships with extended family significantly predicted the occurrence of a major affective episode.[103]

## ROLE OF FAMILY IN THE MANAGEMENT OF PSYCHIATRIC PATIENTS: PROTECTIVE ROLE

### Role of family in treatment

The biopsychosocial model was conceptualized as a medical model that allowed for mutual interaction between the biological and the psychosocial aspects of a person’s life.[104] Modern biological research has elucidated how psychosocial factors influence gene expression and how psychosocial treatments change brain activity.[105,106] More recently, the concept of ”the social brain" has been put forward as a unifying model for how the environment shapes brain development.[107] In these models, the family environment is the most immediate psychosocial milieu. Hence family plays an immense role in the management of psychiatric patients.

Sethi enumerated some unique abilities of family which can be exploited therapeutically in the treatment of mentally ill.[1] These include the ability to: fulfill the physical, spiritual and emotional needs of its members; be sensitive to needs of the family members; communicate effectively; provide support, security and encouragement; initiate and maintain growth, produce relationships and experiences within and without the family; maintain and create constructive and responsible relationships; grow with and through children; accept help when appropriate and also be capable of self help; perform family roles flexibly; have mutual respect for the individuality of family members; use a crisis or seemingly injurious experience as a means of growth; have concern for family unity, loyalty and inter-family cooperation.

Family-based intervention is the most significant contribution of family research to psychiatric practice. The focus of family interventions, to date, has been to build a relationship with caregivers based on understanding and empathy, focusing on the strengths of caregivers and assisting them in identifying community resources, interventions to promote medication compliance, interventions to promote early identification of relapse and swift resolution of the crises, guiding families to reduce social and personal disability, guiding families to reframe expectations and moderate the affect in the home environment, guiding families to improve vocational functioning of the patient, emotional support to caregivers and development of self-help groups for mutual support and networking among families.[108] Successful family intervention reduces rates of relapse and improves quality of life for patients with schizophrenia, bipolar disorder, major depression, borderline personality disorder, and alcoholism.[109–113]

Among studies of family psycho-education for schizophrenia (addressing optimal medication management and addressing the family’s feelings of loss), there is a remarkable consistency of effects on rates of relapse, with minimum reductions of about 50% over the rates for control groups. In 11 of the most rigorous designed and conducted studies, with an average study duration of 19.7 months, the average rate of relapse for the groups receiving family psycho education was 27%, compared to the rate for control groups of 64%.[114] Pfammatter *et al*.[115] conducted a review of three meta-analyses[116–118] of psycho-educational family therapy and a new metanalysis of the 31 most methodologically robust available randomized controlled trials involving over 3,500 clients. They found that compared to medication alone, multi-modal programs which included psycho-educational family therapy and anti-psychotic medication led to lower relapse and rehospitalization rates, and improved medication adherence. One to two years after treatment, the average effect sizes across these four meta-analyses for relapse and rehospitalization rates were 0.32 and 0.48 respectively. The effect size for medication adherence was 0.30. This indicates that the average case treated with family therapy showed better medication adherence than 62% of those who did not receive family therapy. In bipolar disorder, the results of family interventions are more varied. Results from five trials show that family therapy alone or in combination with interpersonal social rhythm therapy (IPSRT) was effective in reducing relapse, and in some instances, rehospitalization in patients with bipolar disorder on maintenance mood stabilizing medication.[119–123] In these trials family therapy was conducted over 21 sessions and included family-based psycho education, relapse prevention, communication and problem solving skills training.[119] On the other hand, trials of other less intensive family-based interventions have not yielded these positive effects.[124–126]

In major depression, the inclusion of a family therapy component led to greater improvement in patients with depression and suicidal ideation than treatment without family therapy.[127] Patients receiving family therapy had significantly greater proportions of patients who improved and significant reductions in interviewer-rated depression and suicidal ideation than patients who were treated without family therapy. Family-based therapies are effective for two of the most debilitating anxiety disorders- agoraphobia with panic disorder and obsessive compulsive disorder. In a review of 12 studies of couple-based treatment for panic disorder and for agoraphobia, Byrne *et al*. concluded that partner-assisted, cognitive-behavioral exposure therapy provided on and individual or group basis led to clinically significant improvement in agoraphobia and panic symptoms for 54 to 86 % of cases.[128] This type of couple therapy was as effective as individually based cognitive-behavioral treatment, widely considered to be the treatment of choice. Family-based approaches have shown to be effective, or in some instances more effective, than individually based cognitive behavior therapy for adults with OCD.[129–132] American Psychiatric Association has recognized the importance of family and includes effective family treatments in its practice guidelines, most notably the practice guideline for schizophrenia.[133] This guideline states that persons with schizophrenia and their families who have ongoing contact with each other should be offered a family intervention, the key elements of which include education about illness, crisis intervention, emotional support and training in how to cope with illness symptoms and related problems. The practice guideline for bipolar disorder and the guideline for major depression also recommend early family involvement and present the known findings about the efficacy of family-based interventions.[134,135]

Many studies have also been done in the Indian context to evaluate the role of family interventions in the management of various psychiatric disorders. Some of the representative Indian studies of family and home treatment in schizophrenia have been shown in Table 1.

**Table 1:** Family intervention studies done in India

Author(s)	Sample	Description	Results
Chacko *et al.* 1967[136]	Mixed group; mainly with schizophrenia	Descriptive study of treatment in a family ward	Benefits from family treatment
Narayanan *et al,* 1972 [137]	104 patients, 76 with schizophrenia	Descriptive study of treatment in a family ward	"Gratifying results" with family treatment
Pai and Kapur, 1982; 1983[138,139]	54 patients with ICD-9 diagnosis of schizophrenia (first episode)	Home treatment by trained nurse vs. hospital treatment	After 6 months, home treatment group significantly improved on symptoms, functioning, family burden
Pai and Roberts, 1983[140]	37 patients with ICD-9 diagnosis of schizophrenia (first episode)	Home treatment by trained nurse vs. hospital treatment	After 2 years home treatment group significantly improved on symptoms only, hospitalized less often
Pai *et al.* 1985[141]	25 patients of chronic schizophrenia,psychosis and epilepsy with > 2 years illness	Home treatment by trained nurse vs. hospital treatment	At 2-year follow-up home treatment group less likely to have been hospitalized; no difference on symptoms, functioning, family burden
Verghese *et al.* 1988[142]	94 caregivers attending a family participation program	Assessment of knowledge and attitudes of carers	Positive change in knowledge and attitudes following attendance
Narayanan *et al.* 1988 [143]	Parents of one hundred and six mentally retarded children	Brief inpatient family intervention	Most parents experienced a favorable change in motivation to train their children and most children had small gains in their skills
Shankar and Menon, 1993[7]	Case reports	Description of a family intervention module	Intervention useful
Sovani, 1993[52]	Caregivers	Descriptive study of a one- day family-psycho education program	Caregivers expressed need for such programs
Ismail Shihabuddeen and Gopinath, 2005[144]	Caregivers of patients with schizophrenia and bipolar disorder	Group meetings conducted for 45 minutes once a month for 17 months	The group meetings led to effective monitoring of the functioning of the individuals, a reduction in the subjective family burden and family distress, a better support system with adequate coping skills and good compliance with treatment programs
Thara *et al.* 2005[145]	Families of persons with schizophrenia	Structured family education utilizing standard evaluation instruments vs. flexible intervention tailored to the needs of the family members.	Most families seemed to prefer the second program, which recorded better attendance and participation. Informal educational sessions with periodic 'across-the-table' re-inforcers may be more effective and practical in the Indian setting.
Kumar and Thomas, 2007[146]	30 patients of alcohol dependence syndrome (DSM-IV) and matched control	Family intervention therapy versus brief supportive psychotherapy as an adjuvant to pharmacotherapy	Family intervention significantly reduced the severity ofalcohol intake, improved the motivation to stop alcohol and changed the locus of control from external to internal over a period of one year
Kulhara *et al.* 2008[147]	76 patients with schizophrenia (DSM-IV) and their caregivers	Psycho education compared with standard outpatient treatment	Structured psycho educational intervention was significantly better than routine out-patient care on several indices including psychopathology, disability, and caregiver-support and satisfaction.

Besides the varied methodology, the common theme of all these interventions is that family interventions contribute to better outcome.

### Role of family in treatment compliance

Nonadherence still remains a major obstacle in the treatment of psychiatric patients. Rates of medication nonadherence among outpatients with schizophrenia have been found to approach 50% during the first year after hospital discharge.[148,149] Scott and Pope reported that one in three persons with bipolar disorder fail to take at least 30 percent of their medication.[150] Medication non adherence in depression varies from 10 to 60% (median 40%) as reported in various studies.[151–153]

Apart from playing a major role in the treatment, families can also play a role in increasing treatment adherence in patients with mental illness. This is done by providing medication to the patient, supervising and monitoring the drug intake, taking the patient to doctor’s chamber at regular intervals, getting serum level of the psychotropics checked so on and so forth.

Studies to enhance treatment adherence among persons with bipolar disorder have found that adherence may be maximized when family members or patients’ significant others are involved, although sole dependence on partners or significant others appears to produce limited results.[154] Samuel and Thyloth in their study of caregivers of 50 consecutively admitted chronic schizophrenia patients in NIMHANS, Bangalore found that in 22 cases (44%), the spouse was responsible for medication management whereas in 10% cases each the responsibility was assumed by siblings and other relatives.[155] Another study has shown that adherence to antipsychotic medication in schizophrenia has a strong positive correlation with total family income, total supervision of medication and percentage visits with attendants.[156]

### Role of family in rehabilitation

The role that families play in the support and care of a relative with a mental illness has gained increasing attention over the past 30 years. The goal of psychosocial rehabilitation is to enable an individual who suffers from mental illness to develop his or her capacities to the fullest extent.[157] In conjunction with individually oriented interventions, psychosocial rehabilitation stresses the importance of environmental factors in the care of people with long-term mental illnesses.[158] Family being the most immediate psychosocial milieu has a significant role in rehabilitation of chronic psychiatric patients.

In has been suggested that in developing countries, caregivers have a major role to play in the resocialization, vocational and social skills training of the patient, not only because of close family ties that exist in these traditional societies, but also because developing countries lack rehabilitation professionals to deliver these services.[159] There is a glaring lack of infrastructure, funds and political support for mental heath care in developing countries. Services are mostly urban-based and therefore relatively inaccessible for a majority of patients who reside in rural areas. In such a situation families have become caregivers of the first and last resort.[160]

A study exploring the role of family support group in psychosocial rehabilitation of mentally ill patients found that family members with a higher level of education are likely to join support groups.[161] Moreover, certain advantages of this kind of group were noted e.g. feeling of togetherness (25%), skills in dealing with the patient at home (20%), emotional support (15%), improvement in relationship with the patient (15%), understanding the problem (15%), reduction in fear and anxiety (10%). In a study by Chatterjee *et al*.[162] community based rehabilitation was found to be better in reducing disability, improving outcome and treatment adherence compared to outpatient care.

### Changes in traditional Indian family

Over the years there have been some diversions from the traditional ideal Indian family and the actual living arrangements vary widely depending on region, social status, and economic circumstances. Although many Indians still live in joint families, which of course deviates in various ways from the ideal joint family, others live in nuclear family set ups and in some other situations a couple lives with their unmarried children, as is the most common pattern in the West. These changes in family structure are amply reflected by the national census data. According to the 1981 census, the population growth was higher than the growth rate of households, a phenomenon which saw a turnaround in 1991 census which showed that the number of households grew at a faster pace than the population and this trend gathered further strength in 2001 census data [Table 2]. This perhaps indicates that nuclearization of families is growing in the society which is more evident in urban areas than in the rural, although happening in both the settings.[163]

**Table 2:** Number of households, average household size and growth by residence 1981-2001

Census Year	Total/urban/rural	Average household size	Decadal growth rate of house holds	Decadal growth rate of population
1981	Total	5.5	19.23	24.65
	Rural	5.6	14.19	19.18
	Urban	5.4	38.43	46.25
1991	Total	5.5	24.68	23.79
	Rural	5.6	20.09	19.89
	Urban	5.3	39.12	36.35
2001	Total	5.3	26.28	21.52
	Rural	5.4	22.31	18.04
	Urban	5.1	37.01	31.06

About 25 years ago, Sethi pointed out that even though the ideal joint family is seldom found, there are often strong networks of kinship ties.[92] Not infrequently, clusters of relatives live very near each other, and are easily available to each other to give and take kinship obligations. Even when relatives cannot actually live in close proximity, they typically maintain strong bonds of kinship and attempt to provide each other with economic help, emotional support, and other benefits.

It has been argued that current sociological and demographic changes taking place in Indian society have a direct bearing on the needs of urban families and hence treatment policies. These changes, which include shift from joint/extended to nuclear family, particularly in urban areas, influx of the traditional female caregivers into the workforce, and migratory movements among the younger generation, have left older caregivers without a second generation of support. It appears that the joint family might not have completely metamorphosed into nuclear units. Most families appear to be in a transitional state where they are structurally nuclear, but still continue to function as joint families do. Members of such "new-order" families may not live under the same roof, but often continue to maintain close ties among themselves.[1,164] Such "functional jointedness" notwithstanding, there has been considerable erosion of traditional support systems and increased stress and pressure on such families, leading to an increased vulnerability to emotional problems and disorders. These social and demographic changes are bound to have an adverse effect on the ability of families to provide care for the mentally ill. It is reflected in the research into the effects of the caregiving experience among Indian families of people with mental illness. With such changes, issues of community care and support for patients without families are beginning to emerge.[80] It is likely that in a country like ours with low income levels and numerous existential stressors, changes in family structure may make the caregiving burden even more onerous. Although there is no data, with the change in scenario with respect to family size, rapid urbanization, cost inflation, increasing trend to spend than to save, it would be interesting to see how all these have affected the kinship.

### What is the implication of changes in family structure for mental health professionals?

It is reasonable to assume that since the process of urbanization and industrialization is occurring at a rapid pace, a substantial number of persons would be experiencing such a transition (from rural to urban and/ or joint to nuclear) in their life time and therefore, being subjected to the stresses in terms of readjustments, reorientation and the making-breaking of human ties. It is known that urbanization is affecting the entire gamut of population especially the vulnerable sections of society - elderly, children and adolescents, and women. The range of disorders and deviancies associated with urbanization are enormous and includes adjustment disorders, depression, sociopathy, substance abuse, alcoholism, crime, delinquency, vandalism, family disintegration, and alienation.[165] Further, now, more and more emphasis is laid on treating the mentally ill in the community for better and easier integration of mentally ill into the society. World wide, there is emphasis on reducing the psychiatric hospital beds and reducing admission into the mental asylums. In a country like India, where the mental healthcare needs have increased but an important resource in the form of family, in the care of the mentally ill subjects is diminishing. Hence, the mental health professionals in India have an important role in promoting the preservation of family, if the needs of mentally ill subjects are to be cared for in a better way.

### What needs to be done?

In a country with more than nine million psychotics and severely mentally retarded, but only one psychiatric bed for 40,000 people, non-institutional, community based mental healthcare is the need of the time. Limited mental health power, which is not going to increase in the required proportion during the coming decades, is an indication to involve non-specialists and paraprofessionals in the care of mentally ill and handicapped. Unlike in the West, almost all the patients and handicapped persons are in the community. If community mental health programs can give know-how and adequate guidance to the family members and voluntary agencies, they can effectively take care of the ill individuals.[4]

At this juncture it would be prudent to examine how the changing structure and functioning of the contemporary Indian family would impact caregiving for people with major mental illness. The World Health Report showed that in several developing countries with low-income economies including India, the government’s share of health expenditure was diminishing, and costs were being increasingly borne by individuals and households.[166] Our policymakers have given importance to the individuals, ignoring the caregivers. Time has come to recognize the contribution of family members who have played an all important role in the treatment and rehabilitation of psychiatric patients.

Access to better treatment for patients, including medications, psychosocial interventions and rehabilitation services is to be ensured. Other measures such as availability of crisis management, provision of legally mandated community treatment to avert hospitalization, and well informed and balanced advocacy are also important. Our mental health policy ought to have the necessary flexibility rather than being dogmatic. Moreover, caregivers need to be supported through active programs of support and guidance rather than being left in the lurch. Family insurance of mentally ill could be a positive step in that direction. In a number of European countries, there exists legislation to provide incentives for families to take care of their patients with long-term disabilities. The mental health professionals should take up this issue with the policy makers.

Family interventions should focus on expanding training to patients and key relatives about wellness recovery, skills training, and task sharing of household and self-care chores. An improvement in these areas is likely to improve the quality of life of people with mental illness and their families. Although the evidence of such positive impact of family interventions in schizophrenia is well documented, such interventions are neither widely used nor appropriately integrated in care plans, and are frequently underfunded. Researchers recommend that in addition to focusing on the symptoms of patients, more attention needs to be given to the mental health and well-being of family caregivers. Adequate information and support have to be extended to the family and caregivers. Given the fundamental differences between India and the West, psychosocial treatments often used so successfully in the West, cannot be directly transposed to the developing world. Hence, there is need to develop a locally applicable theory of rehabilitation and test models of intervention based on this theory.

There has been a rapid propagation of support, self-help, or mutual support groups for family members of persons with severe psychiatric disabilities in the West and to some extent in our country. The families need information, support, knowledge and specific suggestions for coping with mentally ill relatives. Caregivers must be encouraged to join such groups so that they can seek mutual help, learn from experience of others, can share their problems etc.

With the institution of family crumbling because of rapid urbanization and industrialization, the society in general and policymakers must be sensitized to realize the importance of fostering the institution of family for the sake of those ill fated mentally ill people who will be the ultimate sufferer because of lack of mental health infrastructure in India. Time has come to take a positive step towards full integration of family in the care of the mentally ill to combat the ill effects of this changing world and make this world a happier place to live in!

## References

[1] Sethi BB (1989). Family as a potent therapeutic force. Indian J Psychiatry.

[2] Bhushan V, Sachdev DR (2006). The Family. In : Introduction to Sociology.

[3] Heitzman J, Worden RL (1995). India: A Country Study.

[4] Chandrashekar CR, Parthasarathy R, Vyas JN, Nathawat SS, Gada M, Razdan VK (2005). Community Psychiatry. Essentials of Postgraduate Psychiatry.

[5] Anon (1983). Innovations in neuropsychiatric services. NIMHANS J.

[6] Kohlmeyer WA, Fernandes X (1963). Psychiatry in India: family approach in the treatment of mental disorders. Am J Psychiatry.

[7] Shankar R, Menon MS (1993). Development of a framework of interventions with families in the management of schizophrenia. Psychosoc Rehabil J.

[8] Gopinath PS, Rao K (1994). Rehabilitation in Psychiatry: An overview. Indian J Psychiatry.

[9] Bhaskaran K (1970). The unwanted patient. Indian J Psychiatry.

[10] Gupta SP, Yadav BS, Bharadwaj RC, Sharma RP (1980). Psychosocial problems of long stay mental patients. Indian J Psychiatry.

[11] (1999). National Human Rights Commission: Quality Assurance in Mental.

[12] Murthy RS, Mukhopadhyay (1992). State of India’s Health. Mental health.

[13] (1975). World Health Organisation. Organisation of mental care in developing countries. TRS.

[14] Chandrasekhar CR, Isaac MK, Kapur RL, Parathasarathy R (1981). Management of priority mental disorders in community. Indian J Psychiatry.

[15] Wig NN, Murthy RS, Harding TW (1981). A model for rural psychiatric services: Raipur Rani experience. Indian J Psychiatry.

[16] (1982). Director General for Health Services. National Mental Health Program for India.

[17] Sethi BB, Chaturvedi PK (1985). A review and role of family studies and mental health. Indian J Soc Psychiatry.

[18] Sinha D (1984). Some recent changes in the Indian family and their implications for socialization. Indian J Soc Work.

[19] Leff J, Wig NN, Bedi H, Menon DK, Kuipers L, Korten A (1990). Relatives’ expressed emotion and the course of schizophrenia in Chandigarh. A two year follow-up of a first contact sample. Br J Psychiatry.

[20] Bharat S, Bharat S (1991). Research on family structure and problems: review, implications and suggestions. Research on Families with Problems in India: Issues and Implications.

[21] Chandrashekar CR, Rao NV, Murthy RS, Bharat S (1991). The chronic mentally ill and their families. Research on Families with Problems in India: Issues and Implications.

[22] Gopinath PS, Sharma PS, Reddy MV (1987). Patients who discontinue day hospitalization-an analysis. Indian J Psychiatry.

[23] Padmavathi R, Rajkumar S, Srinivasan TN (1998). Schizophrenic patients who were never treated. A study in an Indian urban community. Psychol Med.

[24] Sharma V, Murthy S, Kumar K, Agarwal M, Wilkinson G (1998). Comparison of people with schizophrenia from Liverpool, England and Sakalwara, Bangalore, India. Int J Soc Psychiatry.

[25] Kulhara P, Chakrabarti S (2001). Culture and schizophrenia and other psychotic disorders. Psychiatr Clin North Am.

[26] Thara R, Henrietta M, Joseph A, Rajkumar S, Eaton WW (1994). Ten-year course of schizophrenia-the Madras longitudinal study. Acta Psychiatr Scand.

[27] Eaton WW, Thara R, Federman B, Melton B, Liang KY (1995). Structure and course of positive and negative symptoms in schizophrenia. Arch Gen Psychiatry.

[28] Pearson V, Lam PC, Lefley HP, Johnson DL (2002). Family Interventions in Mental Illness: International Perspectives. On their own: caregivers in Guangzhou, China.

[29] Shankar R, Rao K, Sartorius N, Leff J, L’opez-Ibor JJ, Maj M, Okasha A (2005). From Burden to Empowerment: The Journey of Family Caregivers in India. Families and Mental Disorders: From Burden to Empowerment.

[30] Dani MM, Thienhaus OJ (1996). Characteristics of patients with schizophrenia in two cities in the US and India. Psychiatr Serv.

[31] Platt S (1985). Measuring the burden of psychiatric illnesson the family: an evaluation of some rating scales. Psychol Med.

[32] Hoenig J, Hamilton MW (1966). The schizophrenic patient in the community and his effect on the household. Int J Soc Psychiatry.

[33] Bulger MW, Wandersman A, Goldman CR (1993). Burdens and gratifications of care giving: appraisal of parental care of adults with schizophrenia. Am J Orthopsychiatry.

[34] Braithwaite V (1992). Caregiver burden: making the concept scientifically useful and policy relevant. Res Aging.

[35] Atkinson JM, Coia DA (1995). Families coping with schizophrenia: a practitioner’s guide to family groups.

[36] Barrowclough C, Sartorius N, Leff J, Lopez Ibor JJ, Okasha A (2005). Families of people with schizophrenia. Families and mental disorders: from burden to empowerment.

[37] Awad AG, Voruganti LN (2008). The burden of schizophrenia on caregivers: a review. Pharmacoeconomics.

[38] Schene AH (1990). Objective and subjective dimensions of family burden: toward an integrative framework for research. Soc Psychiatry Psychiatr Epidemiol.

[39] Kuipers E, Paul E, Bebbington PE (2005). Research on burden and coping strategies in families of people with mental disorders: problems and perspectives. Families and mental disorders: from burden to empowerment.

[40] Fadden G, Bebbington P, Kuipers L (1987). The burden of care: the impact of functional psychiatric illness on the patient’s family. Br J Psychiatry.

[41] Lau DY, Pang AH (2007). Care giving experience for Chinese caregivers of persons suffering from severe mental disorders. Hong Kong J Psychiatry.

[42] Onwumere J, Kuipers E, Bebbington P, Dunn G, Fowler D, Freeman D (2008). Care giving and illness beliefs in the course of psychotic illness. Can J Psychiatry.

[43] Gautam S, Nijhawan M (1984). Burden on families of schizophrenic and chronic lung disease patients. Indian J Psychiatry.

[44] Sreeja I, Gupta S, Rakesh L, Singh MB (2009). Comparison of burden between family caregivers of patients having schizophrenia and epilepsy. Internet J Epidemiol.

[45] Chakrabarti S, Kulhara P (1999). Family burden of caring for people with mental illness. Br J Psychiatry.

[46] Chakrabarti S, Raj L, Kulhara P, Avasthi A, Verma SK (1995). A comparison of the extent and pattern of family burden in affective disorders and schizophrenia. Indian J Psychiatry.

[47] Kiran CN (2004). Burden and coping in caregivers of men with alcohol and opioid dependence. Unpublished thesis, submitted to PGIMER.

[48] Nehra R, Chakrabarti S, Kulhara P, Sharma R (2006). Family burden and its correlates among caregivers of schizophrenia and bipolar affective disorder. J Mental Health Human Behav.

[49] Chadda RK, Singh TB, Ganguly KK (2007). Caregiver burden and coping.A prospective study of relationship between burden and coping in caregivers of patients with schizophrenia and bipolar affective disorder. Soc Psychiatry Psychiatr Epidemiol.

[50] Kalra H, Nischal A, Trivedi JK, Dalal PK, Sinha PK (2009). Extent and determinants of burden of care in Indian families: a comparison between obsessive-compulsive disorder and schizophrenia. Int J Soc Psychiatry.

[51] Shaji KS, Smitha K, Lal KP, Prince MJ (2003). Caregivers of people with Alzheimer’s disease: a qualitative study from the Indian 10/66 Dementia Research Network. Int J Geriatr Psychiatry.

[52] Sovani A (1993). Understanding schizophrenia: a family psychoeducational approach. Indian J Psychiatry.

[53] Vijayalakshmi B, Ramana KV (1987). Use of culture-specific coping structures by the families of mentally ill patients. Indian J Soc Work.

[54] Mahendru RK (1996). Mental morbidity and personality pattern in the spouses of schizophrenic patients. Indian J Psychiatry.

[55] Girish K, Pratima M, Isaac MK (1991). Drug treatment in schizophrenia: Issues of comparability and costs. Indian J Psychiatry.

[56] Sarma PG (2000). General hospital psychiatry: Cost of one visit. Indian J Psychiatry.

[57] Chisholm D, Sekar K, Kumar KK, Saeed K, James S, Mubbashar M (2000). Integration of mental health care into primary care.Demonstration cost-outcome study in India and Pakistan. Br J Psychiatry.

[58] Grover S, Avasthi A, Chakrabarti S, Bhansali A, Kulhara P (2005). Cost of care of schizophrenia: A study of Indian out-patient attenders. Acta Psychiatr Scand.

[59] Deshpande SN (2005). Perspective from a general hospital psychiatric unit. Indian J Psychiatry.

[60] Thara R (2005). Perspective from an NGO. Indian J Psychiatry.

[61] Lazarus R, Folkman S (1984). Stress, appraisal and coping.

[62] Lazarus RS (1991). Emotion and adaptation.

[63] Budd RJ, Oles G, Hughes IC (1998). The relationship between coping style and burden in the carers of relatives with schizophrenia. Acta Psychiatr Scand.

[64] Magliano L, Fadden G, Economou M, Xavier M, Held T, Guarneri M (1998). Social and clinical factors influencing the choice of coping strategies in relatives of patients with schizophrenia: results of the BIOMED I study. Soc Psychiatry Psychiatr Epidemiol.

[65] Scazufca M, Kuipers E (1999). Coping strategies in relatives of people with schizophrenia before and after psychiatric admission. Br J Psychiatry.

[66] Magliano L, Fadden G, Economou M, Held T, Xavier M, Guarneri M (2000). Family burden and coping strategies in schizophrenia: 1-year follow-up data from the BIOMED I study. Soc Psychiatry Psychiatr Epidemiol.

[67] Aggarwal A, Avasthi A, Kumar S, Grover S (2009). Experience of care giving in schizophrenia: A study from India. Int J Soc Psychiatry.

[68] Nehra R, Chakrabarti S, Kulhara P, Sharma R (2005). Caregiver coping in bipolar disorder and schizophrenia: A re-examination. Soc Psychiatry Psychiatr Epidemiol.

[69] Ochoa S, Haro JM, Usall J, Autonell J, Vicens E, Asensio F (2005). NEDES group. Needs and its relation to symptom dimensions in a sample of outpatients with schizophrenia. Schizophr Res.

[70] Grinshpoon A, Ponizovsky AM (2008). The relationships between need profiles, clinical symptoms, functioning and the well-being of inpatients with severe mental disorders. J Eval Clin Pract.

[71] Foldemo A, Bogren L (2002). Need assessment and quality of life in outpatients with schizophrenia: a 5-year follow-up study. Scand J Caring Sci.

[72] Bengtsson-Tops A, Hansson L (1999). Clinical and social needs of schizophrenic outpatients living in the community: the relationship between needs and subjective quality of life. Soc Psychiatry Psychiatr Epidemiol.

[73] Wiersma D, Nienhuis FJ, Giel R, Slooff CJ (1998). Stability and change in needs of patients with schizophrenic disorders: a 15- and 17-year follow-up from first onset of psychosis, and a comparison between objective’ and subjective’ assessments of needs for care. Soc Psychiatry Psychiatr Epidemiol.

[74] Hansson L, Björkman T, Svensson B (1995). The assessment of needs in psychiatric patients.Interrater reliability of the Swedish version of the Camberwell Assessment of Needs instrument and results from a cross-sectional study. Acta Psychiatr Scand.

[75] Pearson V (2008). Who cares for the caregivers? Families and schizophrenia in Hong Kong. Hong Kong J Psychiatry.

[76] Graap H, Bleich S, Herbst F, Scherzinger C, Trostmann Y, Wancata J (2008). The needs of carers: a comparison between eating disorders and schizophrenia. Soc Psychiatry Psychiatr Epidemiol.

[77] Kulhara P, Avasthi A, Grover S, Sharan P, Sharma P, Malhotra S (2009). Needs of Indian schizophrenia patients: an exploratory study from India. Soc Psychiatry Psychiatr Epidemiol.

[78] Chadda RK, Pradhan SC, Bapna JS, Singhal R, Singh TB (2000). Chronic psychiatric patients: an assessment of treatment and rehabilitation-related needs. Int J Rehabil Res.

[79] Pradhan SC, Sinha VK, Singh TB (1999). Psycho-social dysfunctions in patients after recovery from mania and depression. Int J Rehabil Res.

[80] Srinivasan N (2000). Families as partners in care.Perspectives from AMEND. Indian J Soc Work.

[81] Neogi R, Chakrabarti S, Grover S (2009). Health care needs of patients with bipolar disorder. Unpublished thesis, submitted to PGIMER.

[82] Shrivastava A, Sakel G, Iyer S (2001). Experience of working with relatives of schizophrenia: evolving caregivers program for advocacy and intervention. Indian J Psychiatry.

[83] Kulhara P, Avasthi A, Sharan P, Sharma P, Malhotra S, Gill S (2001). Assessment of needs of patients of schizophrenia. Indian J Psychiatry.

[84] Nagaswami V, Valecha V, Thara R, Rajkumar S, Menon MS (1985). Rehabilitation needs of schizophrenic patients: a preliminary report. Indian J Psychiatry.

[85] Larson JE, Corrigan P (2008). The stigma of families with mental illness. Acad Psychiatry.

[86] Ping Tsao CI, Tummala A, Roberts LW (2008). Stigma in mental health care. Acad Psychiatry.

[87] Struening EL, Perlick DA, Link BG, Hellman F, Herman D, Sirey JA (2001). The extent to which caregivers believe most people devalue consumers and their families. Psychiatr Serv.

[88] Kadri N, Manoudi F, Berrada S, Moussaoui D (2004). Stigma impact on Moroccan families of patients with schizophrenia. Can J Psychiatry.

[89] Guberman N, Maheu P, Maillé C (1992). Women as family caregivers: Why do they care?. Gerontologist.

[90] Evans AS, Bullard DM, Solomon MH (1961). The family as a potential resource in the rehabilitation of chronic schizophrenic patient: A study of 60 patients and their families. Am J Psychiatry.

[91] Chen FP, Greenberg JS (2004). A positive aspect of care giving: The influence of social support on care giving gains for family members of relatives with schizophrenia. Community Ment Health J.

[92] Sethi BB (1983). Family and Psychiatry. Indian J Psychiatry.

[93] Johnson JG, Cohen P, Kasen S, Smailes E, Brook JS (2001). Association of maladaptive parental behavior with psychiatric disorder among parents and their offspring. Arch Gen Psychiatry.

[94] Cicchetti D, Beeghly M, Cicchetti D, Beeghly M (1987). Symbolic development in maltreated youngsters: An organizational perspective. Atypical Symbolic Development.

[95] Millon T, Davis RD, Cicchetti D, Cohen DJ (1995). The development of personality disorders. Risk, Disorder and Adaptation.

[96] Baumrind D (1971). Current patterns of parental authority. Dev Psychol Monogr.

[97] Steinberg L, Mounts NS, Lamborn SD, Djornbusch SM (1991). Authoritative parenting and adolescent adjustment across varied ecological niches. J Res Adolesc.

[98] Teicher MH, Samson JA, Polcari A, McGreenery CE (2006). Sticks, stones, and hurtful words: relative effects of various forms of childhood maltreatment. Am J Psychiatry.

[99] Dube SR, Anda RF, Felitti VJ, Croft JB, Edwards VJ, Giles WH (200). Growing up with parental alcohol abuse: exposure to childhood abuse, neglect, and household dysfunction. Child Abuse Negl.

[100] Rao U, Weissman MM, Martin JA, Hammond RW (1993). Childhood depression and risk of suicide: a preliminary report of a longitudinal study. J Am Acad Child Adolesc Psychiatry.

[101] Butzlaff RL, Hooley JM (1998). Expressed emotion and psychiatric relapse. Arch Gen Psychiatry.

[102] Heru AM (2006). Family Psychiatry: From Research to Practice. Am J Psychiatry.

[103] Stefos G, Bauwens F, Staner L, Pardoen D, Mendlewicz J (1996). Psychosocial predictors of major affective recurrences in bipolar disorder: A 4-year longitudinal study of patients on prophylactic treatment. Acta Psychiatr Scand.

[104] Engel GL (1982). The biopsychosocial model and medical education: Who are to be the teachers?. N Engl J Med.

[105] Tienari P, Wynne LC, Sorri A, Lahti I, Läksy K, Moring J (2004). Genotype-environment interaction in schizophrenia-spectrum disorder: Long-term follow-up study of Finnish adoptees. Br J Psychiatry.

[106] Roffman JL, Marci CD, Glick DM, Dougherty DD, Rauch SL (2005). Neuroimaging and the functional neuroanatomy of psychotherapy. Psychol Med.

[107] Gardner R (2005). The social brain. Psychiatr Ann.

[108] Murthy RS (2003). Family interventions and empowerment as an approach to enhance mental health resources in developing countries. World Psychiatry.

[109] McFarlane WR, Link B, Dushay R, Marchal J, Crilly J (1995). Psychoeducational multiple family groups: four-year relapse outcome in schizophrenia. Fam Process.

[110] Miklowitz DJ, Simoneau TL, George EL, Richards JA, Kalbag A, Sachs-Ericsson N (2000). Family-focused treatment of bipolar disorder: 1-year effects of a psychoeducational program in conjunction with pharmacotherapy. Biol Psychiatry.

[111] Miller IW, Keitner GI, Ryan CE, Solomon DA, Cardemil EV, Beevers CG (2005). Treatment matching in the posthospital care of depressed patients. Am J Psychiatry.

[112] Gunderson JG, Berkowitz C, Ruiz-Sancho A (1997). Families of borderline patients: a psychoeducational approach. Bull Menninger Clin.

[113] O’Farrell TJ, Murphy CM, Stephan SH, Fals-Stewart W, Murphy M (2004). Partner violence before and after couples based alcoholism treatment for male alcoholic patients: the role of treatment involvement and abstinence. J Consult Clin Psychol.

[114] Baucom DH, Shoham V, Mueser KT, Daiuto AD, Stickle TR (1998). Empirically supported couple and family interventions for marital distress and adult mental health problems. J Consult Clin Psychol.

[115] Pfammatter M, Junghan UM, Brenner HD (2006). Efficacy of psychological therapy in schizophrenia: conclusions from meta-analyses. Schizophr Bull.

[116] Pharoah F, Mari J, Rathbone J, Wong W (2006). Family intervention for schizophrenia. Cochrane Database of Systematic Reviews.

[117] Pilling S, Bebbington P, Kuipers E, Garety P, Geddes J, Orbach G (2002). Psychological treatments in schizophrenia: I. Meta-analysis of family intervention and cognitive behavior therapy. Psychol Med.

[118] Pitschel-Walz G, Leucht S, Bäuml J, Kissling W, Engel RR (2001). The effect of family interventions on relapse and rehospitalization in schizophrenia - a metaanalysis. Schizophr Bull.

[119] Miklowitz DJ, Goldstein MJ (1990). Behavioral family treatment for patients with bipolar affective disorder. Behav Modif.

[120] Miklowitz DJ, George EL, Richards JA, Simoneau TL, Suddath RL (2003). A randomized study of family focused psychoeducation and pharmacotherapy in the outpatient management of bipolar disorder. Arch Gen Psychiatry.

[121] Miklowitz DJ, Richards JA, George EL, Frank E, Suddath RL, Powell KB (2003). Integrated family and individual therapy for bipolar disorder: results of a treatment development study. J Clin Psychiatry.

[122] Miklowitz DJ, Otto MW, Frank E, Reilly-Harrington NA, Wisniewski SR, Kogan JN (2007). Psychosocial treatments for bipolar depression: A 1-year randomized trial from the Systematic Treatment Enhancement Program. Arch Gen Psychiatry.

[123] Rea MM, Tompson MC, Miklowitz DJ, Goldstein MJ, Hwang S, Mintz J (2003). Family-focused treatment versus individual treatment for bipolar disorders and suicide disorder: results of a randomized clinical trial. J Consult Clin Psychol.

[124] Clarkin JF, Carpenter D, Hull J, Wilner P, Glick I (1998). Effects of psychoeducational intervention for married patients with bipolar disorder and their spouses. Psychiatr Serv.

[125] Clarkin JF, Glick ID, Haas GL, Spencer JH, Lewis AB, Peyser J (1990). A randomized clinical trial of inpatient family intervention, V: Results for affective disorders. J Affect Disord.

[126] Miller IW, Solomon DA, Ryan CE, Keitner GI (2004). Does adjunctive family therapy enhance recovery from bipolar I mood episodes. J Affect Disord.

[127] Miller IW, Keitner GI, Ryan CE, Solomon DA, Cardemil EV, Beevers CG (2005). Treatment matching in the posthospital care of depressed inpatients. Am J Psychiatry.

[128] Byrne M, Carr A, Clarke M (2004). The efficacy of couples based interventions for panic disorder with agoraphobia. J Fam Ther.

[129] Renshaw KD, Steketee G, Chambless DL (2005). Involving family members in the treatment of OCD. Cogn Behav Ther.

[130] Grunes M, Neziroglu F, McKay D (2001). Family involvement in the behavioral treatment of obsessive-compulsive disorder: a preliminary investigation. Behav Ther.

[131] Mehta M (1990). A comparative study of family-based and patient-based behavioral management in obsessive compulsive disorder. Br J Psychiatry.

[132] Van Noppen B, Steketee G, McCorkle BH, Pato M (1997). Group and multifamily behavioral treatment for obsessive compulsive disorder: A pilot study. J Anxiety Disord.

[133] Lehman AF, Lieberman JA, Dixon LB, McGlashan TH, Miller AL, Perkins DO (2004). Practice guideline for the treatment of patients with schizophrenia. Am J Psychiatry.

[134] (2002). American Psychiatric Association. Practice guideline for the treatment of patients with bipolar disorder. Am J Psychiatry.

[135] (2000). Practice guideline for the treatment of patients with major depressive disorder (revision). American Psychiatric Association. Am J Psychiatry.

[136] Chacko R (1967). Family participation in the treatment and rehabilitation of the mentally ill. Indian J Psychiatry.

[137] Narayanan HS, Embar P, Reddy GN (1972). Review of treatment in a family ward. Indian J Psychiatry.

[138] Pai S, Kapur RL (1982). Impact of treatment intervention on the relationship between dimensions of clinical psychopathology, social dysfunction and burden on the family of Psychiatric patients. Psychol Med.

[139] Pai S, Kapur RL (1983). Evaluation of home-care treatment of schizophrenic patients. Acta Psychiatr Scand.

[140] Pai S, Roberts EJ (1983). Follow-up study of schizophrenic patients initially treated with home care. Br J Psychiatry.

[141] Pai S, Channabasavanna SM, Nagarajaiah, Raghuram R (1985). Home care for chronic mental illness in Bangalore. An experiment in prevention of repeated hospitalization. Br J Psychiatry.

[142] Verghese A (1988). Family participation in mental health care – the Vellore experiment. Indian J Psychiatry.

[143] Narayanan HS, Girimaji SR, Gandhi DH, Maruthai RK, Rao MP, Nardev G (1988). Brief in-patient family intervention in mental retardation. Indian J Psychiatry.

[144] Ismail Shihabuddeen TM, Gopinath PS (2005). Group meetings of caretakers of patients with schizophrenia and bipolar mood disorder. Indian J Psychiatry.

[145] Thara R, Padmavati R, Lakshmi A, Karpagavalli P (2005). Family education in schizophrenia: A comparison of two approaches. Indian J Psychiatry.

[146] Suresh Kumar PN, Thomas B (2007). Family intervention therapy in alcohol dependence syndrome. Indian J Psychiatry.

[147] Kulhara P, Chakrabarti S, Avasthi A, Sharma A, Sharma S (2009). Psychoeducational intervention for caregivers of Indian patients with schizophrenia: A randomised-controlled trial. Acta Psychiatr Scand.

[148] Babiker IE (1986). Noncompliance in schizophrenia. Psychiatr Dev.

[149] Weiden PJ, Olfson M (1995). Cost of relapse in schizophrenia. Schizophr Bull.

[150] Scott J, Pope M (2002). Self-reported adherence to treatment with mood stabilizers, plasma levels, and psychiatric hospitalization. Am J Psychiatry.

[151] Demyttenaere K, Haddad P (2000). Compliance with antidepressant therapy and antidepressant discontinuation symptoms. Acta Psychiatr Scand Suppl.

[152] Hotopf M, Hardy R, Lewis G (1997). Discontinuation rates of SSRIs and Tricyclic antidepressants: a metaanalysis and investigation of heterogenecity. Br J Psychiatry.

[153] Lingam R, Scott J (2002). Treatment non-adherence in affective disorder. Acta Psychiatr Scand.

[154] van Gent EM, Zwart FM (1991). Psychoeducation of partners of bipolar-manic patients. J Affect Disord.

[155] Samuel M, Thyloth M (2002). Caregivers’ roles in India. Psychiatr Serv.

[156] Warikoo N, Chakrabarti S, Grover S (2008). Treatment adherence in patients of schizophrenia on second-generation antipsychotic medications. Unpublished thesis, submitted to PGIMER.

[157] Fine SB (1980). Psychiatric treatment and rehabilitation: What’s in a name?. J Natl Assoc Priv Psychiatr Hosp.

[158] Liberman RP, Phipps CC, Menninger WW, Hannah GT (1987). Innovative treatment and rehabilitation techniques for the chronically mentally ill. Chronic Mental Patient.

[159] Leggatt M (2002). Families and mental health workers: The need for partnership. World Psychiatry.

[160] Phillips MR, Pearson V (1994). Rehabilitation interventions in urban communities. Br J Psychiatry Suppl.

[161] Ponnuchamy L, Mathew BK, Mathew S, Udayakumar GS, Kalyanasundaram S, Ramprasad D (2005). Family support group in psychosocial rehabilitation. Indian J Psychiatry.

[162] Chatterjee S, Patel V, Chatterjee A, Weiss HA (2003). Evaluation of a community-based rehabilitation model for chronic schizophrenia in rural India. Br J Psychiatry.

[163] (2001). Census 2001: Data Highlights. http://www.censusindia.gov.in/Data_Products/Data_Highlights/Data_Highlights_link/datahighlights_hh1_2_3.pdf.

[164] Gore MS (1968). Urbanisation and Family Change in India.

[165] Trivedi JK, Sareen H, Dhyani M (2008). Rapid urbanization - Its impact on mental health: A South Asian perspective. Indian J Psychiatry.

[166] (2001). The World Health Report 2001. Mental health: New understanding, new hope.

